# Application of Multimodal Navigation together with Fluorescein Angiography in Microsurgical Treatment of Cerebral Arteriovenous Malformations

**DOI:** 10.1038/s41598-017-05913-w

**Published:** 2017-11-01

**Authors:** Shiyu Feng, Yanyang Zhang, Zhenghui Sun, Chen Wu, Zhe Xue, Yudong Ma, Jinli Jiang

**Affiliations:** Department of Neurosurgery, PLA General Hospital, Beijing, 100853 China

## Abstract

This study aimed to explore the clinical applications of multimodal navigation combined with indocyanine green (ICG) fluorescein angiography in microsurgical treatment of cerebral arteriovenous malformations (AVMs). We retrospectively collected 52 patients with AVMs. Assisted by anatomic image, we reestablished three-dimensional structure using preoperative functional magnetic resonance imaging (fMRI) and Diffusion tensor imaging (DTI). The operation for lesion resection was finished under the assistance of neuro-navigation. ICG fluorescein angiography was performed for 16 of the study subjects, meanwhile, FLOW800 was used to rebuild blood vessel color visual image. Brain angiography was performed 1 week after the operation to check residual malformations. The patients’ status was estimated by Modified Rankin Scale score. Of the AVMs, 92.3% (48/52) were totally removed, without severe side events. Among the patients, fluorescein angiography was carried out up to 58 times for 16 cases. All of these 16 cases were confirmed with malformations and 14 of them had draining vein. The total resection rate of these 16 cases reached 100%, and the occurrence rate of postoperative complications was not significantly increased. During the operation of lesion resection, the application of multimodal navigation could effectively protect functional cortex and conduction pathway.

## Introduction

Cerebral arteriovenous malformation (AVM) is a congenital disease owing to cerebral vascular abnormalities in fetus period. In cases with this disorder, abnormal artery is interlinked with other vein directly regardless of the status of capillary bed, leading to malformed vascular mass with expanded vein. Consequently, the disturbance will be triggered in local cerebral hemodynamics, thus showing correlated clinical signs and symptoms^1–3^. Microsurgical treatment is the most complete and reliable treatment for AVM at present. The measure can directly remove malformations, entirely eliminate the risk of rupture, and recover blood circulation in brain tissue^4^. However, the application of microsurgery for cerebral AVM, especially for AVMs in eloquent regions, remains a great challenge due to high risk of complications^5^. Preoperative risk assessment is pivotal for the safety and efficacy of microsurgical treatments among cerebral AVM cases. With the development of technology, neuro-imaging and intra-operative electrophysiology are introduced to microsurgery for AVMs. Under their guidance, the safety of microsurgery for cerebral AVM is considerably improved, contributing to satisfactory effects for patients^6,7^. Indocyanine green (ICG) fluorescein angiography is a real-time intraoperative tool to provide detailed images for vascular architecture, flow direction, and blood flow transit, which can obviously improve the efficacy and safety of microsurgery for AVM as well^8^. However, the application value of ICG fluorescein angiography in microsurgical strategies for cerebral AVMs remains unclear.

This study retrospectively analyzed the data of brain AVMs patients treated with microsurgery together with intra-operative navigation in the department of neurosurgery in PLA General Hospital from June 2009 to January 2015, and explored the applications of intra-operative multimodal navigation and ICG fluorescein angiography in microsurgery using FLOW800 software.

## Materials and Patients

### Selection of cases

A total of 74 patients with brain arteriovenous malformations received microsurgery in the department of neurosurgery in PLA General Hospital from June, 2009 to January, 2015. There were 9 subtentorial cerebel and brain stem AVMs, and 65 supratentorial cerebral AVMs. Among the 65 cases with supratentorial cerebral AVMs, 13 with cerebral hernia resulting from cerebral AVM rupture bleeding received emergency operation while the other 52 were treated with multimodal navigation. Therefore, these 52 patients were selected as our study subjects, containing 35 males and 17 females with an average age of 34.5 (ranging from 4~62 years old), meanwhile 4 cases under 16 years old. Their disease course ranged from 6 hours to 11 years, with an average time of 4.6 months. Moreover, 6 cases had microsurgery history, while 7 underwent radiotherapy and 12 had taken endovascular treatment. In terms of clinical manifestations, 39 cases had headache, 6 cases demonstrated the defect of field vision, 3 cases developed partial aphasia, 8 cases experienced limb weakness or sensory dysfunction and 14 cases suffered preoperative epilepsy. According to Spetzler-Martin grade, 7 cases were classified into grade I, 17 patients were at grade II, 26 cases were at grade III and grade IV included 2 cases (Table 1).

**Table 1:** Spetzler-Martin grade and other characteristics for the 52 patients with AVMs.

Characteristic	No. of Patients (%)
**Size**	
<3 cm	12 (23.08%)
3–6 cm	38 (73.08%)
>6 cm	2 (3.85%)
**Pattern of venous drainage**	
superficial	36 (69.23%)
deep	3 (5.77%)
both	13 (25.00%)
**Location**	
eloquent site	24 (46.15%)
non-eloquent site	28 (53.85%)
**Spetzler-Martin grade**	
I	7 (13.46%)
II	17 (32.69%)
III	26 (50.00%)
IV	2 (3.85%)

The study procedures were approved by the Ethics Committee of PLA General Hospital and 52 patients all signed the written informed consent before the study. The methods were in accordance with the approved guidelines. All authors reviewed the results and approved the final version of the manuscript.

### Microsurgery

**Figure 1:**
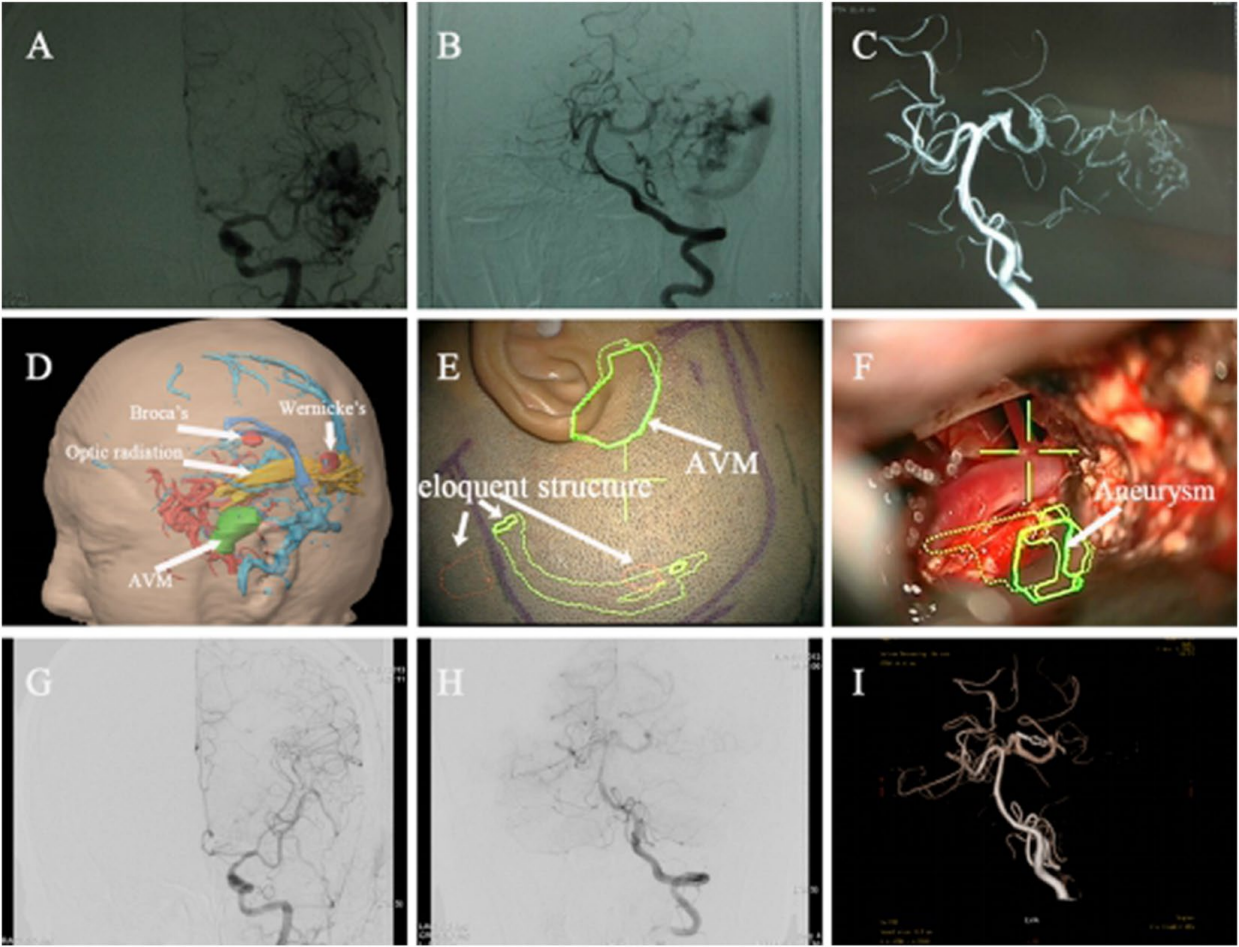
Preoperative DSA (**A**,**B**) demonstrates feeding artery and draining vein in the left temporal AVM; three-dimensional restructure (**C**) shows that left posterior cerebral artery aneurysm has merged into AVM (shown by arrow); Preoperative planning (**D**) indicates the three dimensional spatial position relationship between lesions and functional configuration; (**E**) displays the projections of lesions and functional configuration in the scalp; (**F**) exhibits the projection of aneurysm marked in intra-operative navigation under the microscope; Postoperative DSA (**G**–**I**) illustrates the whole removal of AVMs and successful occlusion of aneurysm.

Preoperative preparation and evaluation: the digital subtraction angiography (DSA) test was performed regularly for each eligible case, showing clear images for feeding artery, draining vein, and the size and location of lesions. According to the results of fMRI, DTI and preoperative preparation, three-dimensional structure was reestablished to demonstrate the location and morphology of lesions, vascular status, functional region and conduction bundle (Fig. 1, 2 **)**. When the distance between malformations and functional cortex or conduction bundle was <20 mm, the lesions were considered to locate in functional areas^9^. EEG monitoring was implemented for patients with epilepsy for 24 hours to discover the location of paradoxical discharge and epileptogenic focus as soon as possible. Preoperative check was evaluated thoroughly, with multiple aspects being taken into consideration. For instance, whether could lesions be removed totally? Would surgery produce acceptable effects? And was it necessary to conduct preoperative embolization for intricate lesions.

**Figure 2:**
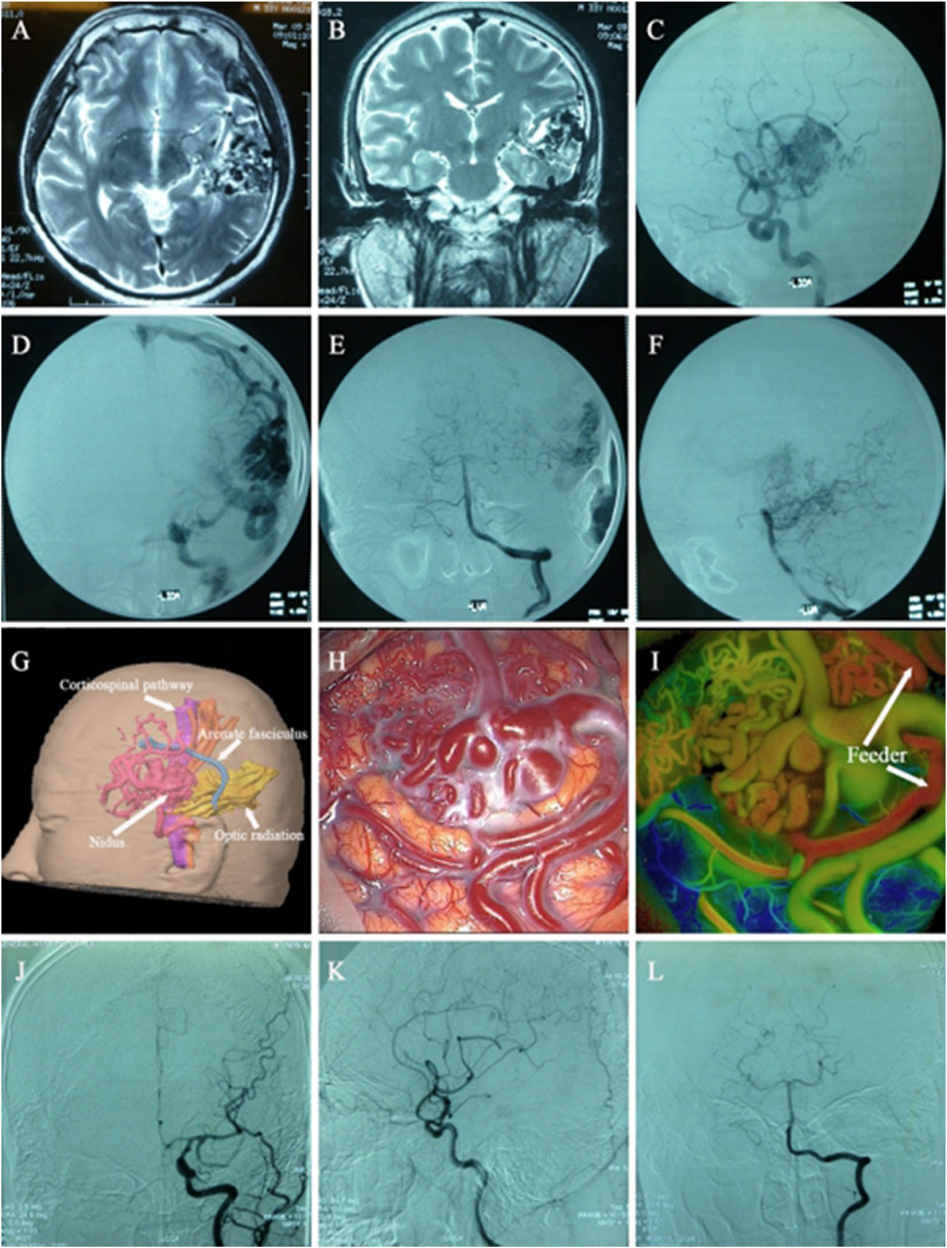
Left temporal lobe AVM and preoperative magnetic resonance (**A,B**) demonstrate the location and size of AVM; Preoperative DSA (**C**–**F**) shows feeding artery and draining vein of AVM; Preoperative planning (**G**) indicates the position relationship between malformations and functional configuration (shown by arrow); (**H**) displays an intra-operative screenshot after the exposure of AVM; (**I**) demonstrates that FLOW800 software could automatically rebuild color visual image, intuitively displaying feeding artery of malformations (shown by arrow); Postoperative DSA (**J**–**L**) illustrate the total resection of AVMs.

Multimodal navigation: General anesthesia and required strict infrared navigation registration were applied in surgery. Appropriate surgical incision and approach were selected based on the projection of lesions, functional cortex and conduction bundle (Fig. 1). Meanwhile, the resection was finished avoiding functional areas as far as possible, with an area little bigger than lesion. Navigation system would manifest the location of malformations along with their significant structures onto microscope (Pentero, Carl-Zeiss, Oberkochen,Germany) during the operation. Different colors under the microscope symbolized diverse structures, which were beneficial to removing malformations and protecting vital functional configuration simultaneously. For lesions near functional areas, especially motor cortex and fasciculi pyramidalis, suspicious feeding artery of malformations would be occluded temporarily, then the risk evaluation was performed. With regards to the possibility of navigation shift, intra-operative magnetic resonance was utilized to correct navigation information, thus obtaining the location of lesions during the whole process. After removing malformations, magnetic resonance was manipulated again to scan whether residual malformations existed. As for patients with epilepsy, cortical electrode was adopted during the operation to locate epileptogenic focus.

**Figure 3:**
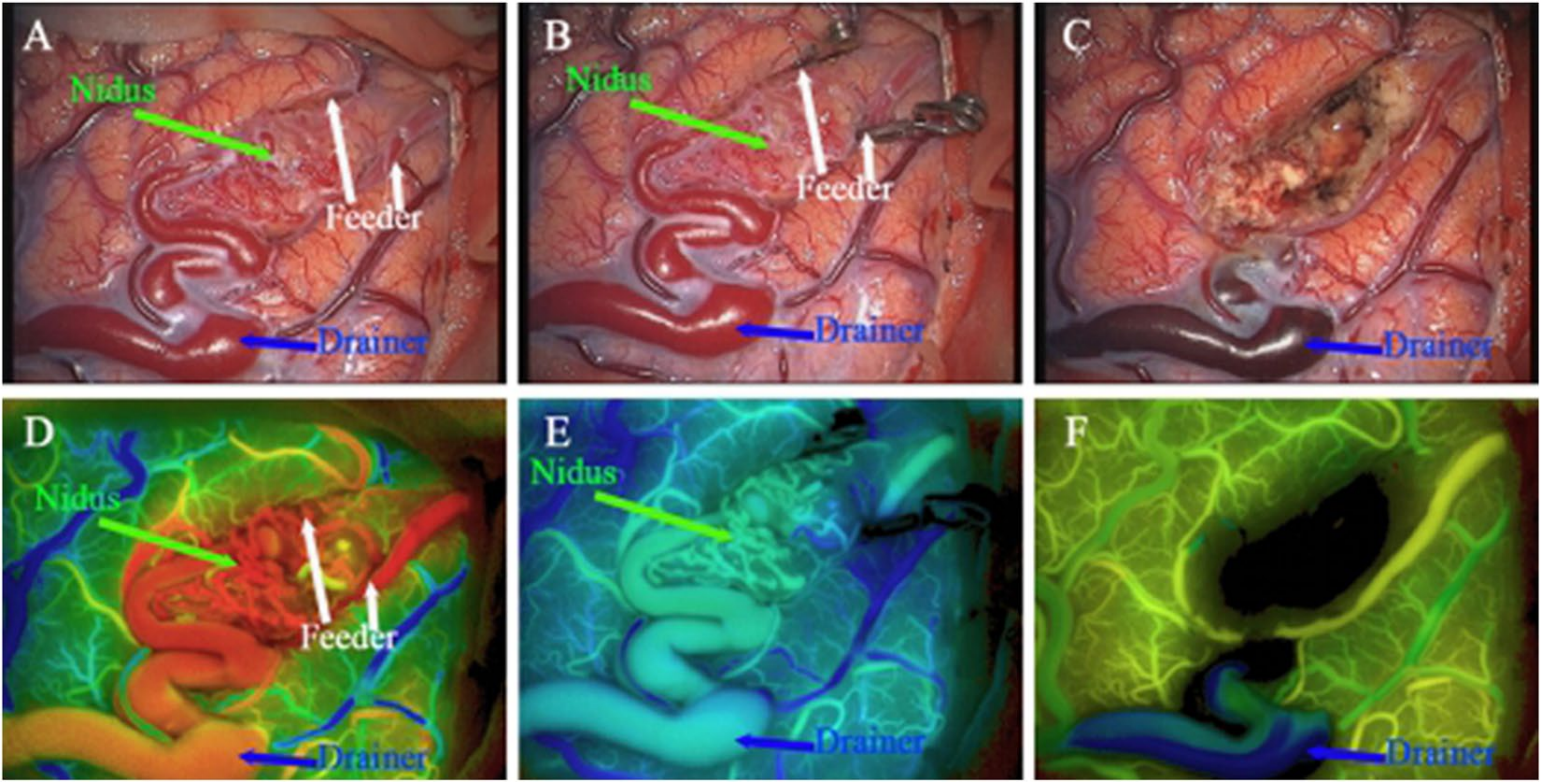
Color visual image offers intuitively images for changes in blood flow, and the operation region was rebuilt based on spontaneous analysis using FLOW800 software: (**A**) color map before clipping feeder. The components of AVM are displayed instinctively; (**B**) color map after clipping feeder. The color deepening of nidus and drainer indicates a decrease in shunt flow; (**C**) color map after AVM resection; (**D**–**F**) statuses before occlusion, after occlusion and after the removal of malformations, respectively.

Intra-operative fluorescein angiography: Lesions were exposed after opening dura mater. Then, surgical field was cleaned up and observed vascular malformations were placed under the microscope which was switched to fluorescein angiography mode with 300 mm focal length, 5X amplification factor and 50% illumination intensity. Fresh ICG solution was compounded (25 mg contrast medium was dissolved in 10 mL sterile saline) with 0.2–0.5 mg/kg single dosage. After the usage of fluorescein, angiography mode of microscope FLOW800, rapid bolus injection of contrast medium was performed through large venous indwelling needle. Once reaching the target area which was irradiated by infrared light in the microscope, ICG began to image, and fluorescein angiography could be observed repeatedly. Blood vessel color visual image of operation region was rebuilt based on spontaneous analysis through FLOW800 software (Carl Zeiss Co, Oberkochen, Germany), which could intuitively demonstrate feeding artery, draining vein of malformations (Fig. 3), and blood flow direction and sequence. Region of interest (ROI) in the strongest fluorescence imaging was then chosen, thus generating the time-intensity curves of various colors and calculating time and slope of 1/2 peak intensity automatically. Subsequently, half-quantitative objective assessment was implemented for the characteristics of hemodynamics in operation region. After feeding artery and draining vein were determined, the feeding artery was clamped with temporary occlusion to perform fluorescein angiography again (15 min or more after the latest angiography); additionally, hemodynamic changes were determined through the combination of the angiography with intra-operative electrophysiologic monitoring.

### Follow-up methods

Brain angiography was applied regularly 1 week after operation to examine whether residual malformations existed or not. If residual malformations were observed, stereotactic radio surgery was recommended, and then MRI along with DSA were operated for the examination regularly. Otherwise, only MRI and DSA were employed every 8~12 month in the follow-up. Modified Rankin Scale score was adopted to evaluate patients’ functional status.

### Statistical analysis

Data analysis was performed with SPSS 18.0 software (SPSS Inc., Chicago, IL, USA). Continuous variables were summarized as average ± SD, and compared using student’s t test. Categorical variables were shown in numbers and percentage, and chi-square test was applied for data analysis. *P* values less than 0.05 was considered to be statistically significant.

## Results

### Intra-operative neuronavigation

Surgery incision was operated for all of the 52 patients, and surgical approach was determined under the assistance of intra-operative navigation. Among these cases, 28 with arteriovenous malformations not covering functional area experienced general navigation while 24 with malformations involving functional area received multimodal navigation surgery. The function cortex and function tracts were identified in intra-operative navigation.Total resection was achieved in 20 of the 24 cases implicating functional areas, who kept intact function; as for the other 4 patients, their arteriovenous malformations were found to have close ties to the function areas where residues existed. For these 4 cases, detailed situations were as follows: Patient 1, malformation was in deep basal ganglia region, and closely related to pyramidal tract, with deformity group in deep venous drainage; Patient 2, residue was reserved, because physiological monitoring operation found that clipping a suspicious deformity would evoke potential decline in motion significantly; Patients 3 and 4, deformities were close to arcuate beam, so intraoperative awakening was not used and appropriate residuals were reserved to preserve function.

### Intra-operative fluorescein angiography

FLOW800 with intra-operative fluorescein angiography was not introduced into our hospital until 2013. Consequently, FLOW800 fluorescein angiography was only applied in 16 patients during operation for a total of 58 times, with an average of 3.6 times per case (ranging 3~6 times). Additionally, 16 times of angiography was carried out before AVMs resection, 23 times during the resection of AVMs after clipping draining artery, and 19 times for malformations which were considered to be removed totally by surgeons. In the resection of malformations, 1~2 feeding arteries were determined in 15 AVMs patients. All of the 16 cases experiencing FLOW800 fluorescein angiography were diagnosed with malformations, and 14 them had draining vein simultaneously (Fig. 3).

We compared the preoperative and postoperative characteristics between patients receiving fluorescein angiography and those without. Analysis results demonstrated similar preoperative characteristics in the two groups (*P* > 0.05 for all). After the occlusion of feeding artery, fluorescein angiography showed that blood flow was reduced or delayed in 16 patients. For the 16 patients with AVMs totally removed, intra-operative fluoresce in angiograph showed no residuals, with a total resection rate of 100%. More importantly, some common complications such as epilepsy and irritability did not occur in the patients after injection with contrast medium. Fluorescein angiography did not show significant effects on postoperative Rankin score (*P* = 0.345), but significantly shorten the postoperative hospital stay (*P* = 0.032) (Table 2).

**Table 2:** The efficacy and safety of fluorescein angiography in microsurgical treatments for patients with AVM.

Indexes	Fluorescein angiography group (n = 16, 30.77%)	Non-fluorescein angiography group (n = 36, 69.23%)	*P*
Gender			0.257
Male	9	26	
Female	7	10	
Average age (years)	36.3 ± 5.5	33.6 ± 7.8	0.321
Course disease (months)	5.3 ± 11.2	4.3 ± 12.1	0.567
Size			0.525
<3 cm	3	9	
3–6 cm	13	25	
>6 cm	0	2	
Pattern of venous drainage			0.786
Superficial	12	24	
Deep	1	2	
Both	3	10	
Location			0.404
Eloquent site	6	18	
Non-eloquent site	10	18	
Spetzler-Martin grade			0.115
I + II	10	14	
III + IV	6	22	
Surgery			0.165
Totally resection	16	32	
Residues	0	4	
Postoperative hospital stay (days)	10.3 ± 2.1	18.6 ± 4.5	0.032
Preoperative epilepsy	5	9	0.562
Postoperative epilepsy	0	2	0.350
Postoperative bleeding	0	3	0.247
Preoperative Rankin score	1.1 ± 0.8	1.2 ± 0.7	0.772
Rankin score (1 week postoperative)	2.4 ± 0.9	2.7 ± 1.0	0.345

### Postoperative complications

All of the patients stayed in hospital for 7~42 days. And postoperative bleeding was observed in 3 cases, containing 1 receiving endoscopic removal of hematoma due to postoperative residuals resulting from intimate relationship between malformations and functional results; 1 with residual malformations which were found in emergency DSA for cerebral vascular finding and needed to be removed after the resection of hematoma in original incision; and 1 with epidural hematoma but without any obvious complications after surgery. What’s more, among the 52 cases, some developed new neurological dysfunctions: 3 patients had hemiplegic paralysis and 2 of them recovered 3~6 months after operation while the other 1 recovered partially; 2 patients got aphasia and recovered completely after half a year; 2 cases with defects in field vision did not recover completely; 2 cases with postoperative hydrocephalus were better after undergoing ventriculo-peritoneal shunt; and 3 cases with new epilepsy after operation gained acceptable effects from correlated drugs.

### Prognosis and follow-up results

The degree of removal of malformations was judged during the operation and 1 week after the operation. Among the 52 cases, 48 (92.3%) achieved total resection, including 21 (87.5%) of the 24 patients with AVMs involving functional areas and 27 (96.4%) of the 28 cases with AVMs not implicating functional areas. The postoperative follow-up lasted 3~70 months, with an average of 22.5 months. Additionally, 4 cases with residual malformations were recommended for stereotactic radio surgery; as a result, residual malformations in 2 of them were blocked, 1 was followed up regularly in the reaction period of radiation therapy and the other 1 died from AVM recurrence and cerebral hernia 3 months after leaving the hospital. Cortical electrical stimulation was adopted to locate epileptogenic focus during the operation for 14 patients with preoperative epilepsy, 12 of whom had no epilepsy postoperatively and the other 2 took oral antiepileptic drugs to control epileptic seizure. The average value of Rankin score was 1.2 before the operation and 2.6 1 week after the operation, and we thought such increase might result from transient postoperative reactions like hydrocephalus. As expected, the score dropped to 1.4 3 months after the operation. In the latest follow-up, 44 cases received telephone follow-up, including 36 cases (81.8%) backing to daily work and study, 5 being able to take care of themselves while 3 under other peoples’ care.

## Discussion

Up to now, several treatments have been developed for treating arteriovenous malformations, like microsurgical resection, endovascular embolization and radiotherapy. The main purpose of these treatments is to avoid bleeding, control epilepsy and prevent neurological dysfunction and progressive deterioration. Stereotactic radiotherapy possibly be effective to treat patients with AVMs diameter below 3.5 cm, which is mainly applied for deep-seated lesions in brain functional areas. Only few patients treated with radiotherapy could recover within 1~3 years, but such technique may lead to some delayed complications like haemorrhage, radioactive brain edema and necrosis^10,11^. Endovascular embolization is primarily applied for smaller malformations, and adopted as an assisting tool for bigger malformations before surgery so as to reduce the risk of intra-operative bleeding^7,12^. Until now, microsurgical resection represents the most complete and reliable treatment for AVMs that can remove AVMs and epileptogenic focus totally and avoid vascular malformation hemorrhage. In our study, 52 AVMs patients were all treated using microsurgery under guidance, and the microsurgical effects were analyzed among these subjects.

Arteriovenous malformations in the 52 patients were removed with the assistance of functional neuro navigation. If lesions were in functional areas, such as motor area, sensory area and language area, or conduction bundles like fibrae pyramidales and bow beam, optic radiation, preoperative reestablishment was necessary. Multimodal Navigation can display the position relationship between disease focus and functional areas or conduction bundles. According to multimodal magnetic images, malformations could be removed completely along their edges, with functional areas and conduction bundles intact. thus minimizing surgical-related adverse effects. In 24 patients, different functional cortices and bundles were involved, so all of the corresponding functional cortices were activated preoperatively and adjacent bundle was traced out as well. Cranial iPlan 2.6 (BrainLab) was utilized to make three dimensional planning before the operation, displaying the relationship of disease focus with activated functional cortex and rebuilt bundles, and calculating the distance between malformations and adjacent functional configuration. Intra-operative images magnetic resonance, CT and intra-operative cortical electrical stimulation were employed to correct the navigation shift caused by brain displacement, judging functional locations more exactly.

Intra-operative multimodal navigation can offer a better understanding not only about vascular structure in patients with AVMs, but also about the location of epileptogenic focuses as well as the relationship between their gyri and critical structures through synthetically anatomizing imaging, white matter conduction bundle, cortical functional areas and intra-operative electrophysiologic monitoring^13^. However, AVMs usually lead to changes in surrounding cerebral blood flow dynamicsin, which account for the deviation in functional cortex from fMRI position^14,15^. Consequently, it is necessary to locate functional areas, particularly the motor area using appropriate approaches during the operation, like electrophysiologic monitoring. For cases with malformations next to motor area or fasciculi pyramidalis, navigation combined with electrophysiologic monitoring can work well, but further locations of language area and visual cortex are still demanded. Focusing on resection AVMs next to motor area and language area, Michael T *et al*.^16^ adopted electrophysiology along with wake-up craniotomy to locate functional areas, with a total resection rate of 67% (8/11). In our study, 21 (87.5%) out of 24 cases involving with functional areas gained full removal. Total resection is an effective therapeutic strategy for patients with AVMs, and residual malformations may increase risk of bleeding. Multimodal navigation can provide detailed information for vascular morphology and location, so with its guidance, the total resection rate can be significantly elevated during microsurgical treatments for patients with AVMs.

Intra-operative ICG fluorescein angiography has been applied for 30 years, mainly for rapid evaluating patency of blood vessels in the Department of Neurosurgery^17^. The application of FLOW 800 combined with microscope has being increased in recent years. This approach half-quantitatively analyzes the distribution of transient hemodynamics in surgical region and colors encoding visualization on the basis of ICG fluorescein angiography. It is characterized by establishing vascular color visual image in surgical region, promptly displaying the direction and sequence of blood flow, drawing time-intensity curves and objectively assessing the hemodynamic status in operation area^18,19^. ICG fluorescein angiography exhibits obvious advantages in microsurgery for AVMs, such as identifying feeding artery and draining vein. After ICG injection through vena cava, feeding artery starts to appear, followed by draining vein. In this work, we only analyzed the data of FLOW800 fluorescein angiography from 16 AVMs patients. And the results showed that 1~2 feeding artery were determined in 15 of these patients, and all of them were confirmed with malformations while 14 of them also had draining vein. Accurate blocking of feeding artery is critical for the safety of surgery. Although the main feeding artery is blocked, other vessels may still supply blood for malformations. Step by step, tiny feeding artery is fulgerized along normal brain tissue on the edge of malformations, and draining vein is not blocked until malformations completely removed. We found that ICG was suitable for the identification of angioarchitecture in superficial AVMs, but to totally expose deeper AVMs in operation area under microscope was not easy. With the guidance of ICG fluorescein angiography, the total resection reached 100%, higher than that without guidance, despite lacking statistical significance. In addition, we also found that ICG fluorescein angiography was safe for patients with AVMs receiving microsurgery. Specifically, the occurrence of postoperative complications, such as bleeding, epilepsy, was not significantly increased after ICG injection. Moreover, the postsurgical hospital stay was significantly shortened among patients accepting ICG fluorescein angiography. However, in our study, only 16 cases received ICG fluorescein angiography. The relatively small sample size might limit the representativeness of the results, and further investigations with larger sample size will be required to estimate the efficacy and safety of ICG fluorescein angiography in microsurgical treatments for patients with AVMs.

**Figure 4:**
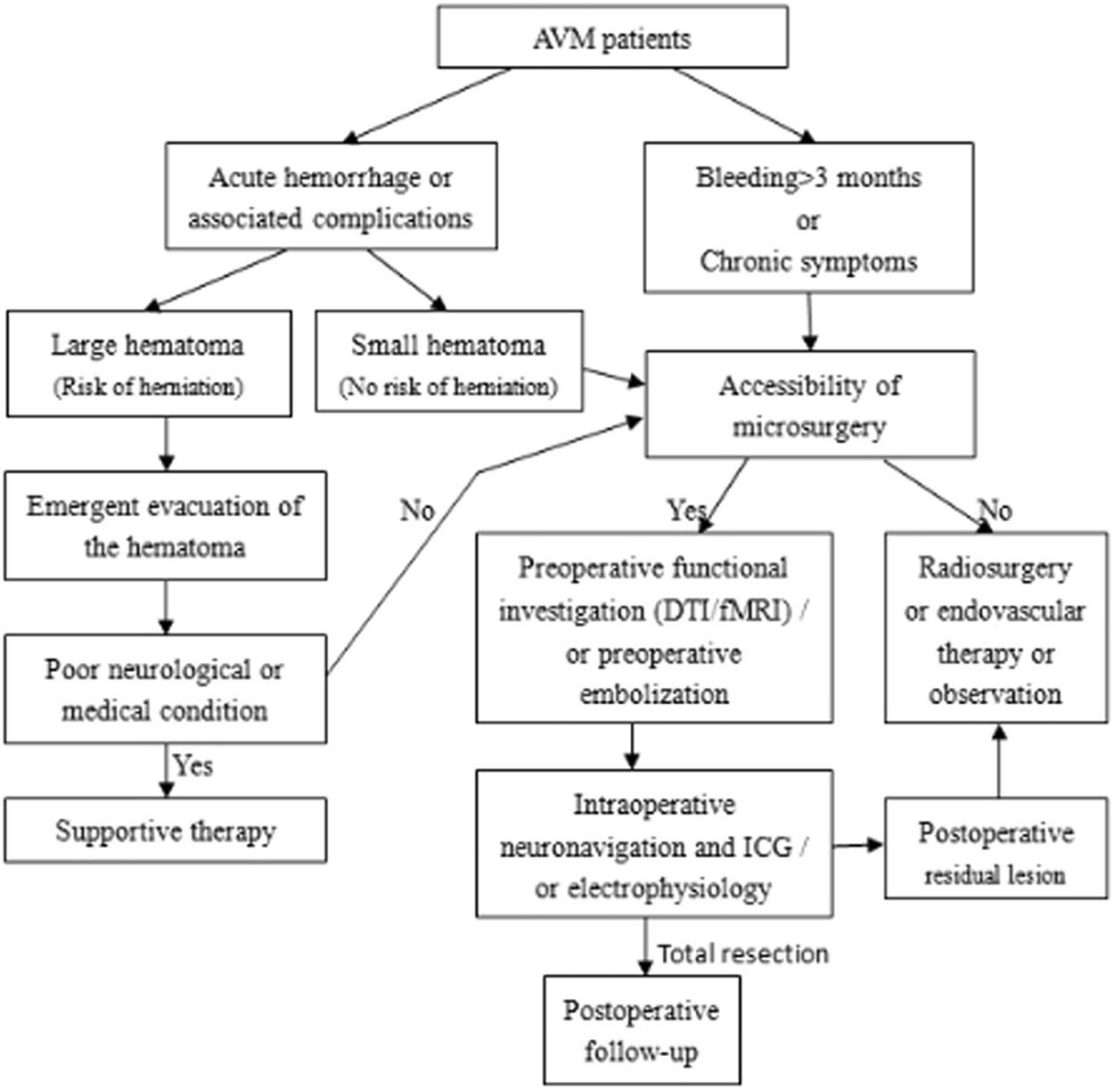
Flow chart for microsurgery management in patients with AVMs according to relevant publications and our experiences. The flow chart included the patients selection, preoperative functional evaluation, and the decision of the extent of surgical resection.

AVMs usually leads to spontaneous rupture, so certain preoperative preparations are essential to handle acute bleeding, previous bleeding and chronic symptom. For patients with extensive preoperative intracranial hemorrhage, AVMs might even result in cerebral hernia and coma. Furthermore, preoperative functional navigation planning was impossible for this condition even if functional areas were involved. If possible, emergency DSA or CTA would be adopted to investigate reasons for bleeding, and accordingly, emergency operation was implemented to remove hematoma while intracranial pressure monitoring was performed, even for patients receiving decompressive craniotomy. Patients with serous nervous dysfunction resulting from acute hemorrhage would recovery well after receiving appropriate and timely treatments. In addition, detailed evaluation of functions would be accomplished before surgical treatment^16^. Based on our clinical experiences, patients would benefit from the intra-operative application of multimodal navigation conjunction with electrophysiologic monitoring in microsurgery, especially for hemorrhage ones without severe nervous dysfunction and those with good operation indication. Various approaches were used for preoperative functional configuration, including fMRI and DTI (Fig. 4). Nonetheless, some patients’ preoperative functions were not assessed because of obvious nervous dysfunction, so stereotaxic radiosurgery were recommended for them to treat smaller lesions while follow-up and observations were adopted for bigger ones. Besides, positive surgical treatments were proposed for younger patients given their higher cerebral tolerance, whereas follow-up and observations were recommended for older ones with functional areas involved so as to lower the risk of cumulative bleeding^12,16^ (Fig. 4). For patients whose malformations showed intimate relationship with functional configuration in preoperative assessment and those failing to finish preoperative estimation due to long-term chronic weakness caused by hemorrhage, positive surgery might further deteriorate nervous dysfunction, and tereotactic radio surgery or conservative treatment was recommended.

In conclusion, microsurgical resection is an effective treatment for patients with cerebral AVM in clinical practice. Preoperative evaluation through brain angiography and regular MRI including fMRI and DTI is pivotal for therapeutic effects and safety of microsurgery. Moreover, the safety of microsurgery can significantly benefit from guidance approaches, such as functional neural navigation, electrophysiologic monitoring and other advanced technologies like FLOW800.
